# 3D-bioprinted bone scaffolds: redefining paediatric orthopaedics reconstruction

**DOI:** 10.1097/MS9.0000000000003887

**Published:** 2025-09-17

**Authors:** Eisha Ali, Sheza Kamran, Asad Ali Ahmed Cheema

**Affiliations:** a Department of Medicine, FMH College of Medicine and Dentistry, Lahore, Pakistan; b International School of Medicine, International University of Kyrgyzstan, Bishkek, Kyrgyzstan

*To the Editor,*

This manuscript has been prepared in compliance with the TITAN Guidelines 2025 for transparency, integrity, and appropriate use of artificial intelligence in scholarly publishing^[1]^.

The intricacies of children’s skeletal growth and development make pediatric orthopedic reconstruction one of the most difficult areas of clinical practice. Despite being widely used, traditional bone grafting techniques have drawbacks such as donor-site morbidity, infection risk, and limited graft availability. However, new developments in tissue engineering, specifically the creation of 3D-printed scaffolds infused with hydroxyapatite and stem cells, present encouraging opportunities to enhance bone regeneration^[2]^.

Contradictory to conventional technology, bioprinting uses medical images to create structures customized for the patient. In pediatric patients, where growth potential and skeletal adaptation are critical, 3D bioprinting creates complex and delicate biomimetic 3D structures that simulate the extracellular matrix preparing high precision multifunctional scaffolds that integrate, remodel, and grow alongside the native bone. Osteogenic differentiation has been confirmed by osteocalcin quantification and alkaline phosphatase staining, further validating scaffold-induced osteogenesis^[3]^. Preclinical studies have shown promising results in terms of vascularization, osteointegration, and mechanical stability of bio-printed constructs offering a paradigm shift in the unique management of congenital bone defects^[4]^.

Insufficient autologous bone grafting, poor graft histocompatibility, and insufficient induced bone regeneration can all be resolved with bone tissue engineering. Since 3D-printed scaffolds contain growth factors that offer a better option for individualized treatment, bone defect repair, and regeneration, which are essential for long-term functional outcomes in developing children, surgeons can expect to perform fewer revision surgeries in addition to more dependable reconstructions^[5]^.

Despite these developments, obstacles such as legal restrictions, exorbitant expenses, and the requirement for long-term safety data restrict procedure translation into clinical practice. In pediatrics, where ensuring growth compatibility and biocompatibility is crucial, ethical considerations are especially pertinent; hence, to bridge the gap between laboratory innovation and bedside application, bioengineers, clinicians, and policymakers must work together in order for clinical practice to adopt this innovation and use it to provide long-lasting care.

## Data Availability

Not applicable – no new data were generated or analyzed in this Letter to the Editor.

## Research registration unique identifying number (UIN)

Not applicable – this Letter to the Editor does not involve human participants and is based on previously published literature.
